# Identification of pyroptosis-related immune signature and drugs for ischemic stroke

**DOI:** 10.3389/fgene.2022.909482

**Published:** 2022-09-27

**Authors:** Shanshan Shi, Qi Zhang, Changda Qu, Yushi Tang, Yewei Qu, Shirong Wen, Ruohan Sun, Yujun Pan

**Affiliations:** ^1^ Department of Neurology, The First Affiliated Hospital, Harbin Medical University, Harbin, China; ^2^ Key Laboratory of Hepatosplenic Surgey Ministry of Education, The First Affiliated Hospital, Harbin Medical University, Harbin, China

**Keywords:** ischemic stroke, pyroptosis, systemic inflammation, immunity, LASSO-cox regression, RNA modification

## Abstract

**Background:** Ischemic stroke (IS) is a common and serious neurological disease, and multiple pathways of cell apoptosis are implicated in its pathogenesis. Recently, extensive studies have indicated that pyroptosis is involved in various diseases, especially cerebrovascular diseases. However, the exact mechanism of interaction between pyroptosis and IS is scarcely understood. Thus, we aimed to investigate the impact of pyroptosis on IS-mediated systemic inflammation.

**Methods:** First, the RNA regulation patterns mediated by 33 pyroptosis-related genes identified in 20 IS samples and 20 matched-control samples were systematically evaluated. Second, a series of bioinformatics algorithms were used to investigate the contribution of PRGs to IS pathogenesis. We determined three composition classifiers of PRGs which potentially distinguished healthy samples from IS samples according to the risk score using single-variable logistic regression, LASSO-Cox regression, and multivariable logistic regression analyses. Third, 20 IS patients were classified by unsupervised consistent cluster analysis in relation to pyroptosis. The association between pyroptosis and systemic inflammation characteristics was explored, which was inclusive of immune reaction gene sets, infiltrating immunocytes and human leukocyte antigen genes.

**Results:** We identified that AIM2, SCAF11, and TNF can regulate immuno-inflammatory responses after strokes via the production of inflammatory factors and activation of the immune cells. Meanwhile, we identified distinct expression patterns mediated by pyroptosis and revealed their immune characteristics, differentially expressed genes, signaling pathways, and target drugs.

**Conclusion:** Our findings lay a foundation for further research on pyroptosis and IS systemic inflammation, to improve IS prognosis and its responses to immunotherapy.

## 1 Introduction

Cerebral stroke is an acute cerebrovascular disease caused by the sudden rupture of blood vessels in the brain or the inability of the blood to reach the brain, like in ischemic or hemorrhagic stroke (Wang et al., 2020). Ischemic stroke (IS) is a general term for necrosis of brain tissues caused by stenosis, occlusion, or insufficient blood supply of the carotid and vertebral arteries to the brain. The incidence of IS is higher than that of hemorrhagic stroke, accounting for 60–70% of the total stroke cases, and is more common in males over 40 years old than in females (Cercy et al., 2018; Liu et al., 2021a). Oxidative free-radical damage, excitatory amino acid toxicity, intracellular calcium overload, inflammation, and apoptosis have been recognized to potentially result in IS. Pyroptosis is reported to participate in IS progression directly through cell death and neuroinflammation (Ye et al., 2020). Therefore, the immune motor mechanism of pyroptosis on IS might be key to revealing IS pathologies, which offers directions to explore new therapies for the patients.

The term “pyroptosis” was initially identified in 2001 when caspase-1-dependent cells died in salmonella-induced macrophages (Boise and Collins, 2001). Further research found that other proinflammatory caspases like caspase-1/3/4/5/11 can also mediate pyroptosis in addition to caspase-1. Thus, pyroptosis has been redefined as gasdermin-mediated programmed necrosis (Ye et al., 2020). During IS progression, inflammasomes activate caspase-1 through an adapter protein associated with apoptosis, whereas they directly bind to lipopolysaccharides to activate caspase-4/5/11 (Tan et al., 2021). The activated caspase-1/4/5/11 specifically cleaves GSDMD (gasdermin-D) into the N-terminal (NT) and the C-terminal (CT) domain; the activated caspase-3 cleaves GSDME (gasdermin-E). The gasdermin-NT of GSDMD and GSDME mediates the formation of plasma membrane pores, leading to cytoplasmic swelling, large bubble formation from the cell membrane, and rapid intracellular content release. Eventually, cell lysis occurs. The mature interleukin-18 (IL-18) and interleukin-1β (IL-1β) are cleaved into biologically active, mature, and proinflammatory cytokines by activated caspase-1 (Liu et al., 2021a; Qing-Zhang. et al., 2021).

According to previous research, the immune system has an intricate impact on the pathophysiological changes that occur after IS, which appear to involve cerebral and systemic inflammation (Masahito. and Yenari Midori, 2015). After occurrence of cell death and brain tissue injury associated with IS, activated microglia and ischemic endothelial cells secrete proinflammatory agents and chemokines, and the accumulation of circulating immune cells starts with the rapid upregulation of adhesion molecules, selectins, and immunoglobin superfamily members (Carlo Domenico. et al., 2020). Leukocytes cause cerebral ischemic injury through different mechanisms (Mathias. et al., 2014; Torres-Aguila Nuria et al., 2019), and cerebral mast cells regulate early ischemic brain swelling and neutrophil accumulation, which is in correlation with severe neurologic damage and indicates an increased mortality risk (Strbian et al., 2006). Similar to neutrophils, lymphocytes have a negative impact on stroke (Masahito. and Yenari Midori, 2015). Thus, preventing infections that exacerbate systemic inflammation and inhibiting neural pathways that trigger inflammatory responses are potential therapeutic targets for IS patients (Tomasz, 2015).

Pyroptosis, known as inflammatory injury, has had few pathological mechanism studies focused on its relation with systemic inflammation of IS. Therefore, we designed an integrative analysis pipeline (Supplementary Figure S1). Here, we first systematically evaluated PRGs’ regulation pattern in IS systematic inflammation. It was found that the PRGs were good differentiators of IS and healthy samples. Then, immune reactivity and infiltrating immunocyte abundance of cells affected by IS showed significant correlations with pyroptosis, suggesting a close association between immune regulation and pyroptosis. Next, IS samples were clustered in 33 PRGs, and three distinct pyroptosis regulation patterns were determined. The different immune characteristics among these patterns were studied, and their biological responses and functions were also compared. Finally, we used the clusterProfiler enrichment analysis to evaluate the DEGs of different patterns. Our findings indicated that pyroptosis has a crucial impact on IS systemic inflammation.

## 2 Materials and methods

### 2.1 Acquisition and pretreatment of data

The data in the present study were inclusive of 20 IS samples and 20 sex- and age-matched healthy controls. The data were reserved in the Gene Expression Omnibus (GEO) database under accession number GSE22255, which was deposited by Krug et al. (2012). According to the manufacturer’s instructions, the gene expression was profiled using the Affymetrix Human Genome U133 Plus 2.0 Array microarrays. We acquired the data using the “GEOquery” R package and preprocessed them according to a previous study. We used log base 10 function transformation to normalize the data. The probes were annotated as gene symbols, and those without matching symbols were excluded. The median value was selected as duplicate gene symbols’ expression. As for the 33 PRGs studied in this study, we referred to the conclusions drawn from previous research studies (Man and Thirumala-Devi, 2015; Wang and Yin, 2017; Ye et al., 2021; Wang et al., 2022). The “empirical Bayes method” in the R package called “limma” (Gentleman Robert et al., 2004; Carey et al., 2005)was applied to calculate the differential genes between IS and normal groups. The expression value was preprocessed by the ‘Normalize Between Arrays’ function in the ‘limma’ package.

### 2.2 Alteration analysis of pyroptosis genes in ischemic stroke and healthy controls

The protein–protein interaction (PPI) network of 33 PRGs was constructed using the Search Tool for the Retrieval of Interacting Genes (STRING) database (Szklarczyk et al., 2019) and then visualized using Cytoscape (Smoot Michael et al., 2011) software. Then, we performed the Pearson correlation coefficient (PCC) in all samples and compared their expressions between IS samples and healthy controls using the “limma” R package, and the cut‐off criteria were *p* < 0.05. Univariate logistic regression was used to identify IS-associated pyroptosis genes with *p* < 0.05 as the cut-off criterion. The LASSO (least absolute shrinkage and selection operator) regression was used for feature selection and dimension reduction. Multivariate logistical regression was used to develop a pyroptosis regulator-related IS classifier. Receiver operating characteristic (ROC) curve analysis was used to evaluate the distinguishing performance of the signature.

### 2.3 Correlation analysis between pyroptosis genes and immune characteristics

Single-sample gene set enrichment analysis (ssGSEA) defines an enrichment score to represent the degree of a gene set’s absolute enrichment in every sample within a given dataset (Barbie David et al., 2009). Here, the specific infiltrating immunocytes and the activity of specific immune reactions were estimated using ssGSEA. We acquired the gene sets used for evaluating the infiltrating immunocytes from a previous study (Shen et al., 2019) and downloaded gene sets related to immune reactions from the ImmPort database (Karlsen and Haabeth, 1998). Then, the enrichment scores of immunocyte abundance and immune reaction activity, and HLA (human leukocyte antigen) genes’ expression were compared using the Wilcox test between IS samples and healthy controls. The correlation of pyroptosis genes with immunocyte fractions, immune reaction activity, and HLA expression was performed using PCC analysis.

### 2.4 Identification of pyroptosis gene regulation patterns

We performed an unsupervised clustering analysis on 33 PRG expressions for identifying their regulation patterns. A consensus clustering algorithm was adopted to assess the cluster numbers and robustness (Rui-Chao. et al., 2019; Zhang et al., 2020). The robustness of classification was guaranteed using the “ConsensusClusterPlus” R package (Wilkerson Matthew and Hayes, 2010) with the aforementioned steps for 1000 iterations. The expressions of 33 PRGs in different regulation patterns were further validated using principal component analysis (PCA).

### 2.5 Identification of DEGs among genes mediated by pyroptosis-related genes

We analyzed samples of distinct PRG regulation patterns by the empirical Bayesian approach of the “limma” R package to determine genes mediated by PRGs among different regulation patterns. *p* < 0.01 was set as the criteria of significant DEGs.

### 2.6 Biological enrichment analysis of distinct pyroptosis-related genes and identification of related drugs

We analyzed PRGs’ biological functions and genes mediated by PRGs through the “clusterProfiler” R package (Yu and Wang, 2012) in GO-BP (Gene Ontology-biological process) enrichment analysis. Biological signaling pathways can reflect biological changes, and KEGG (Kyoto Encyclopedia of Gene and Genomes) pathway analysis was used in this study. Enriched pathways of commonly shared DEGs in different regulation patterns were analyzed using the “clusterProfiler” R package. Additionally, to identify the latent target drugs of IS, the list of drug–gene interactions was obtained from the Drug–Gene Interaction Database (DGIdb) (Cotto Kelsy et al., 2018). As a web resource, it can consolidate disparate data sources which describe gene druggability and drug–gene interactions. Thus, the PRGs of different regulation patterns were, respectively, intersected with the list of drug–gene interactions to obtain latent target drugs for IS.

### 2.7 Statistical analyses

All statistical analyses were conducted using R software (version 4.0.5). We used the *t*-test to compare two groups and the Kruskal–Wallis test to compare more than two groups. Spearman and distance correlation analyses were used to calculate correlation coefficients. The correlation between pyroptosis patterns and clinical phenotypes was analyzed using the chi-squared test. The statistical significance threshold was set to *p* < 0.05 (two-tailed).

## 3 Results

### 3.1 Identification of DEGs between healthy and ischemic stroke samples

**FIGURE 1:**
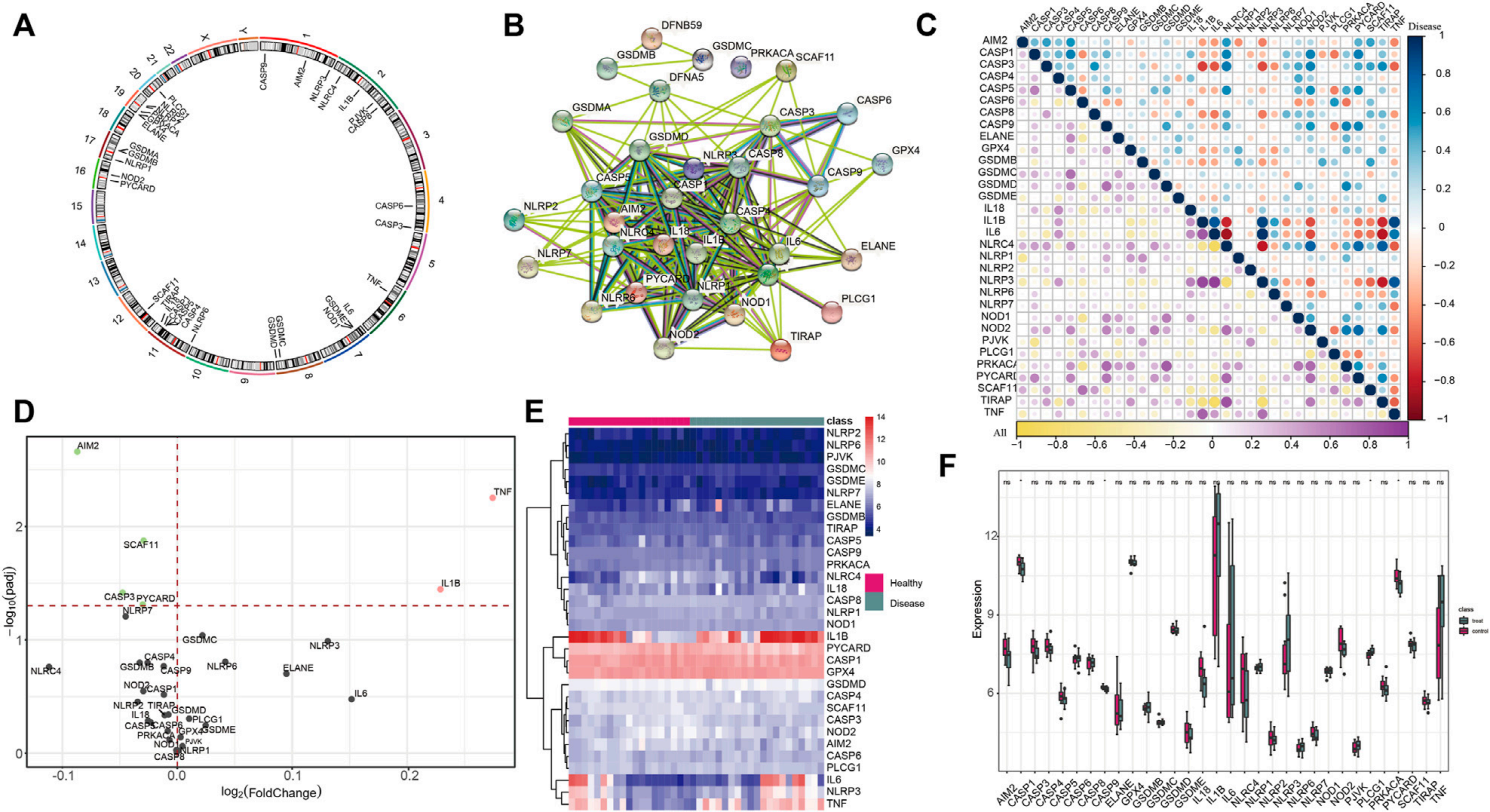
Identification of DEGs between healthy and IS samples. **(A)** Distribution of 33 PRGs on chromosomes. **(B)** Thirty-three PRG protein–protein interactions are presented. **(C)** Correlations among the expression of pyroptosis genes in all healthy and IS samples. **(D)** Volcano plot shows the summary of expression-changing information of 33 PRGs between healthy and IS samples. **(E)** Heatmap plot demonstrates the transcriptome expression status of 33 PRGs between healthy and IS samples. Blue: low expression level; red: high expression level. **(F)** Box plot demonstrates the transcriptome expression status of 33 PRGs between healthy and IS samples.

There were 33 PRGs involved in the study, and their distribution on chromosomes is shown in Figure 1A. To depict the transcriptome interactions between these PRGs, we constructed the PPI network (Figure 1B). The analysis showed close correlations among them (Figure 1C). Then, we compared the expression levels of 33 PRGs in 20 normal and 20 IS samples and determined 6 DEGs (*p* < 0.01). Among them, four genes (AIM2, CASP3, SCAF11, and PYCARD) were downregulated, and two genes (TNF and IL1B) were upregulated and abundant in the IS group (Figure 1D). Next, the 33 PRGs were divided into four groups on the basis of their expression levels (Figure 1E), excluding those not showing noticeable change, suggesting that they might not perform a paramount function in IS (Figure 1F). We also validated these genes in another two datasets (GSE16561 and GSE1954425) which shows that AIM2, IL-1B, and PYCARD are significant differentially expressed. CASP3 and SCAF11 were also differentially expressed but to some extent (Supplementary Figures S3,S4). TNF has been proved to be highly expressed in the blood of patients with cerebral infarction in many previous studies (Lin et al., 2010; Liu et al., 2021b). All the aforementioned findings provide certain support for our research results.

### 3.2 Pyroptosis-related genes can well distinguish between healthy and ischemic stroke samples

**FIGURE 2:**
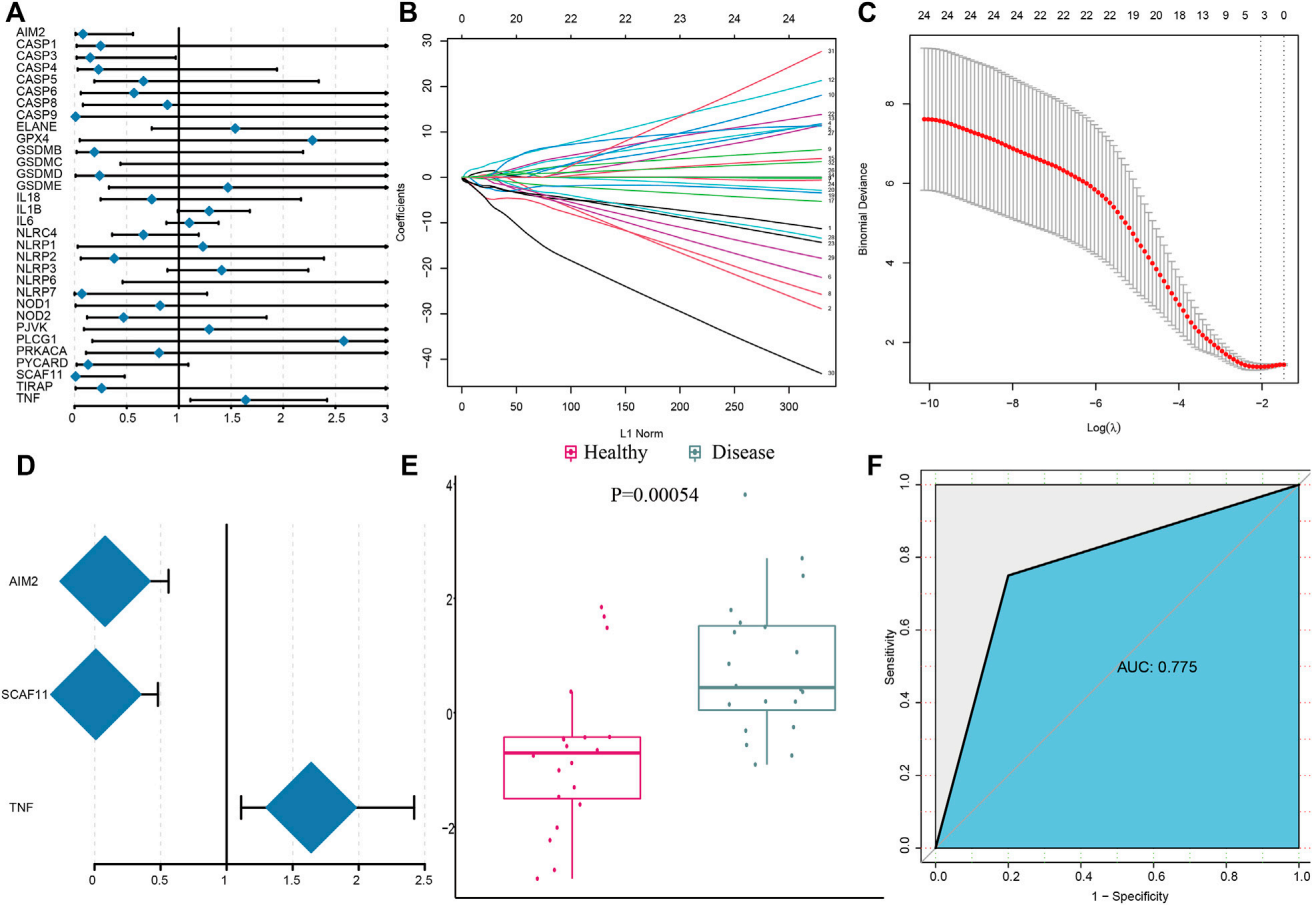
Pyroptosis genes can well distinguish healthy and IS samples. **(A)** Univariate logistic regression investigated the relationship between PRGs and IS. **(B)** Least absolute shrinkage and selection operator (LASSO) coefficient profiles of 33 PRGs. **(C)** Ten- fold cross-validation for tuning parameter selection in the LASSO regression. The partial likelihood of deviance is plotted against log(λ), where λ is the tuning parameter. Partial likelihood deviance values are shown, with error bars representing SE. The dotted vertical lines are drawn at the optimal values by minimum criteria and 1-SE criteria. **(D)** Distinguishing signature with three PRGs was developed by multivariate logistic regression, and the risk scores for IS were calculated. **(E)** Risk distribution between healthy and IS samples, where IS samples has a much higher risk score than healthy samples. **(F)** Discrimination ability for healthy and IS samples by PRGs was analyzed using the ROC curve and evaluated by the AUC value.

We employed a series of bioinformatics algorithms to investigate PRGs’ contribution to IS pathogenesis. Univariate logistic regression identified four pyroptosis regulators in relation to IS (Figure 2A) and then LASSO regression was conducted on these four pyroptosis regulators for dimension reduction and feature selection to exclude the unimportant regulators (Figures 2B,C). The results demonstrated that three PRGs were crucial for IS development. Multivariate logistic regression developed a classifier to distinguish between healthy and IS samples (Figure 2D). The classifier made up of three PRGs distinguished between healthy and IS samples on the basis of risk scores and showed that IS had a much higher pyroptosis risk score than healthy samples (Figure 2E). The PCA result demonstrated a diverse pyroptosis regulator expression pattern between IS and healthy samples (Figure 2F). The ROC curve demonstrated that the three pyroptosis regulators had a positive impact on classifying IS and healthy samples, expounding their crucial impact on IS development (Figure 2G).

### 3.3 Pyroptosis-related genes are associated with the immune characteristics of ischemic stroke

**FIGURE 3:**
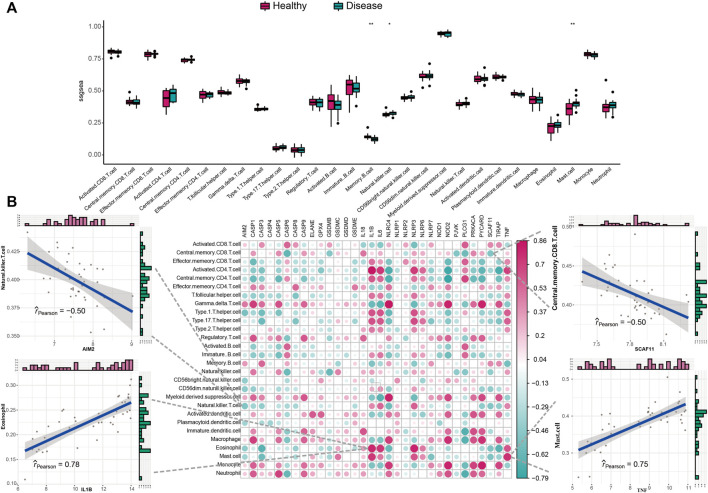
Correlation between infiltrating immunocytes and pyroptosis genes. **(A)** Difference in the abundance of each infiltrating immunocyte between healthy and IS samples. **(B)** Dot-plot demonstrated the correlations between each infiltrating immunocyte type and each PRG.

**FIGURE 4:**
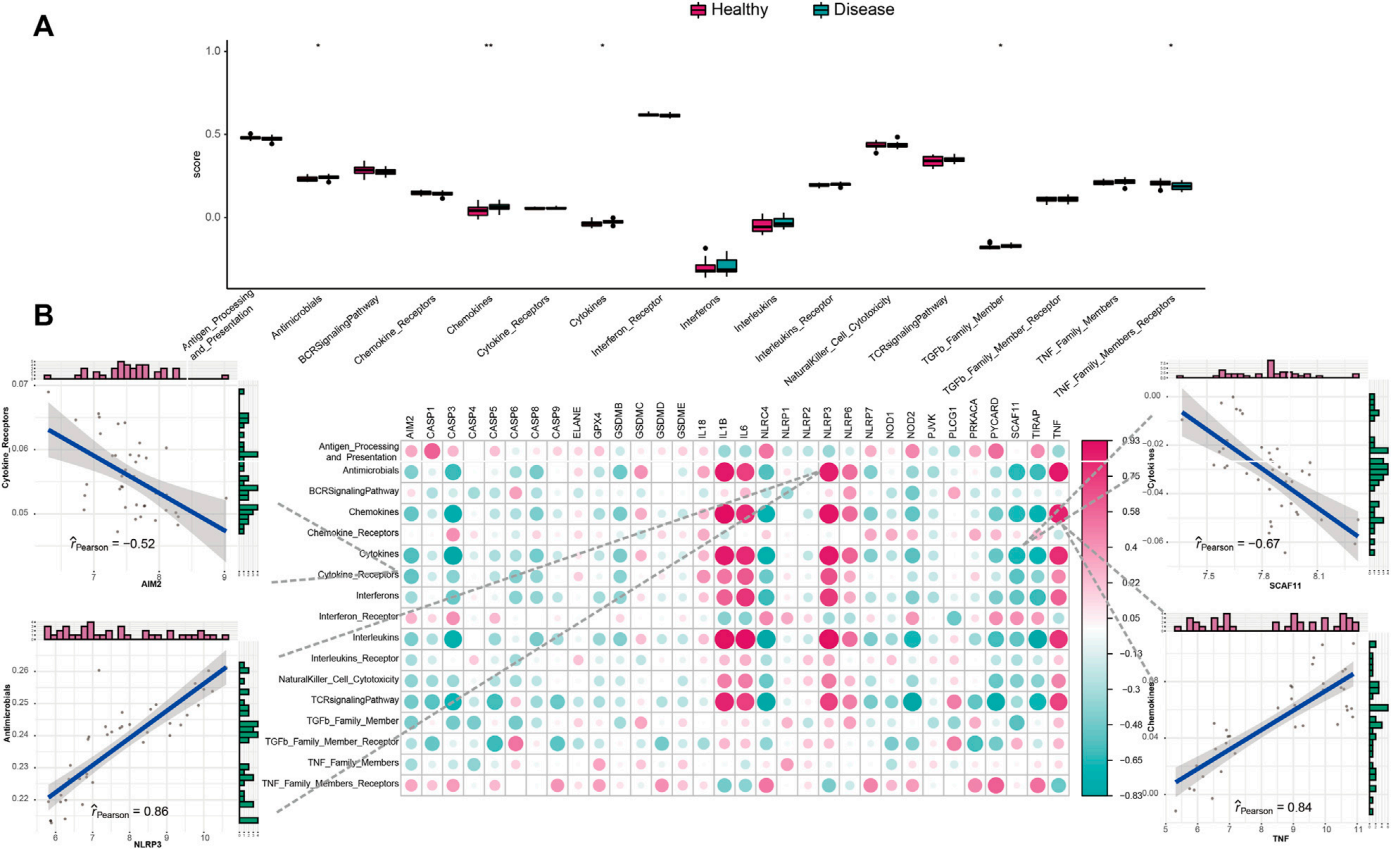
Correlation between immune reaction gene sets and pyroptosis genes. **(A)** Difference in the activity of each immune reaction gene set between healthy and IS samples. **(B)** Dot-plot demonstrated the correlations between each dysregulated immune reaction gene set and each PRG.

We studied the biological correlation between pyroptosis regulators and systemic inflammation by performing correlation analysis for mal-adjusted pyroptosis regulators with the immune reaction genome, infiltrating immunocytes, and HLA expression. Differences in infiltrating cell abundance of 28 immune microenvironments were revealed between healthy and IS samples (Figure 3A). Several infiltrating immune cell portions were altered in IS, such as memory B cell, nature killer (NK) cell, and mastocyte. The correlation analysis showed that pyroptosis-related genes closely correlated with many immune cells (Figure 3B). For example, NK T-cell abundance was negatively correlated with AIM2, indicating that their increased infiltration in IS was closely related to AIM2 expression. Eosinophil and activated CD4^+^ T-cell abundance were positively correlated with IL1B, indicating that their increased infiltration in IS was closely related to IL1B expression. Mastocytes were positively correlated with TNF, indicating that the increased infiltration of mastocytes in IS was closely correlated with its expression. The immune reactions and HLA in IS were also analyzed (Figure 4A and Supplementary Figure S2A). The differences in the activity of every immune reaction genome between IS and healthy samples, as well as the immune responses to increased or decreased immune cell infiltration in IS, such as the activities of chemokines and cytokines, were presented. These results indicated that AIM2 and SCAF11 were negatively correlated with cytokine receptors and their activity, respectively. NLRP3 and TNF were positively correlated with antimicrobials and chemokines, respectively (Figure 4B). Similarly, we also explored HLA expression and found insignificant correlations (Supplementary Figure S2B).

### 3.4 Pyroptosis-related gene modification patterns mediated by 33 regulators in ischemic stroke

**FIGURE 5:**
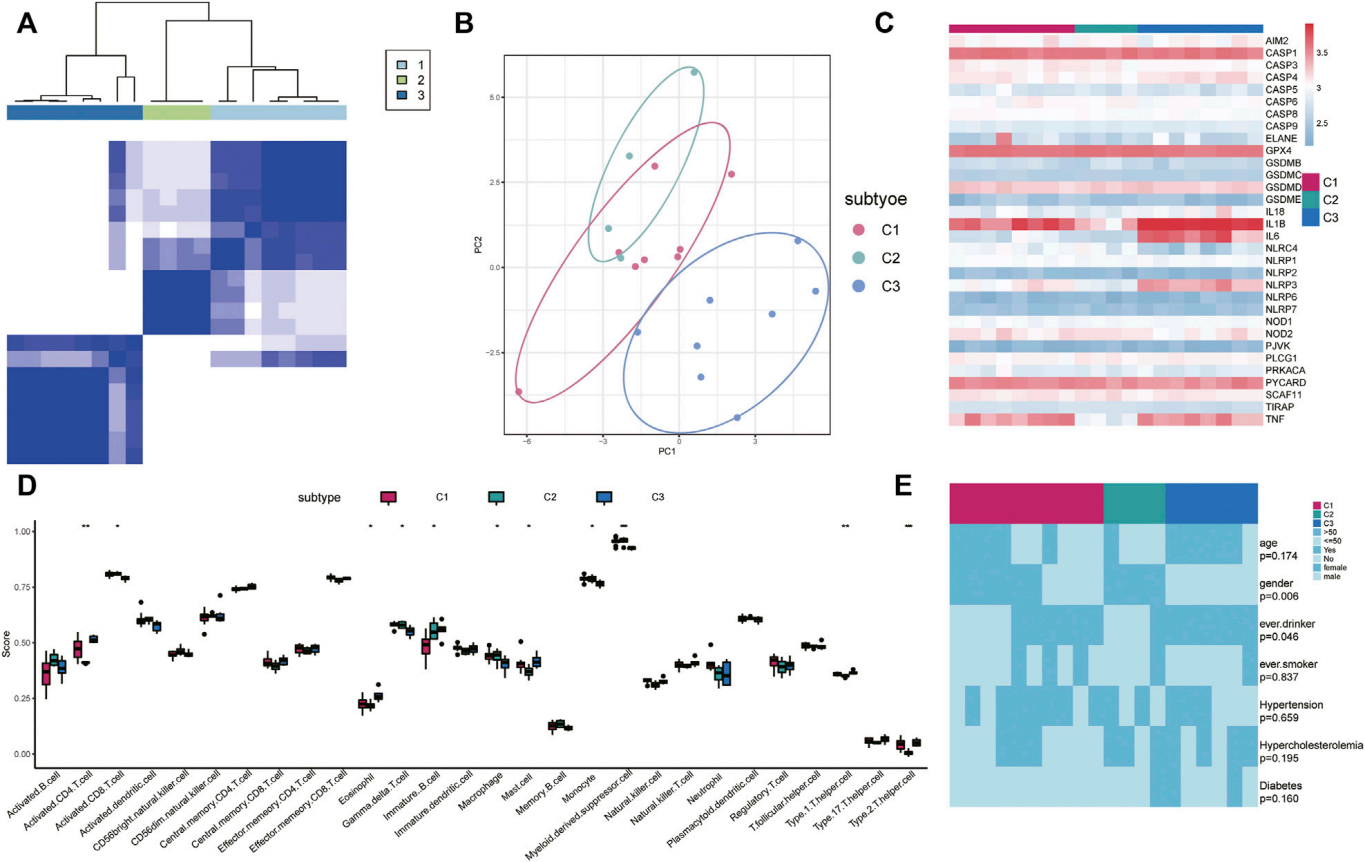
Identification of different pyroptosis expression patterns. **(A)** Heatmap of the matrix of co-occurrence proportions for IS samples. Blue: low-expression level; red: high-expression level. **(B)** Principal component analysis for the transcriptome profiles of three pyroptosis subtypes, showing a remarkable difference in the transcriptome between different modification patterns. **(C)** Expression status of 33 PRGs in the three pyroptosis subtypes. **(D)** Unsupervised clustering of 33 PRGs in the three patterns **(E)** Comparison of age, sex, smoking, alcohol consumption, hypertension, hypercholesterolemia, and diabetes and IS type among three pyroptosis regulation patterns. The heatmap illustrates the association of different clinical characteristics with the three subtypes.

We conducted an unsupervised consensus clustering analysis for IS samples based on the expressions of 33 PRG regulators to investigate gene modification patterns of pyroptosis in IS (Figure 5A). Three distinct subtypes of IS were identified with qualitatively different expressions of the 33 PRG regulators including eight samples in subtype-1, four samples in subtype-2, and eight samples in subtype-3 (Figure 5B). Distinct expression patterns of PRGs in the three subtypes are shown in Figure 5C. Meanwhile, we found that the infiltration levels of many immune cells were different among the three subtypes. For example, activated CD8^+^ T cells were higher in subtypes C1 and C2 but the lowest in subtype C3 (Figure 5D). Eosinophils were lower in subtypes C1 and C2 while being the highest in subtype C3. In addition, differences in gender (*p* = 0.006) and alcohol consumption (*p* = 0.046) were also distinct among the clinical characteristics of different regulatory patterns (Figure 5E).

### 3.5 Biological characteristics and potential drugs of three pyroptosis-related gene modification patterns

**FIGURE 6:**
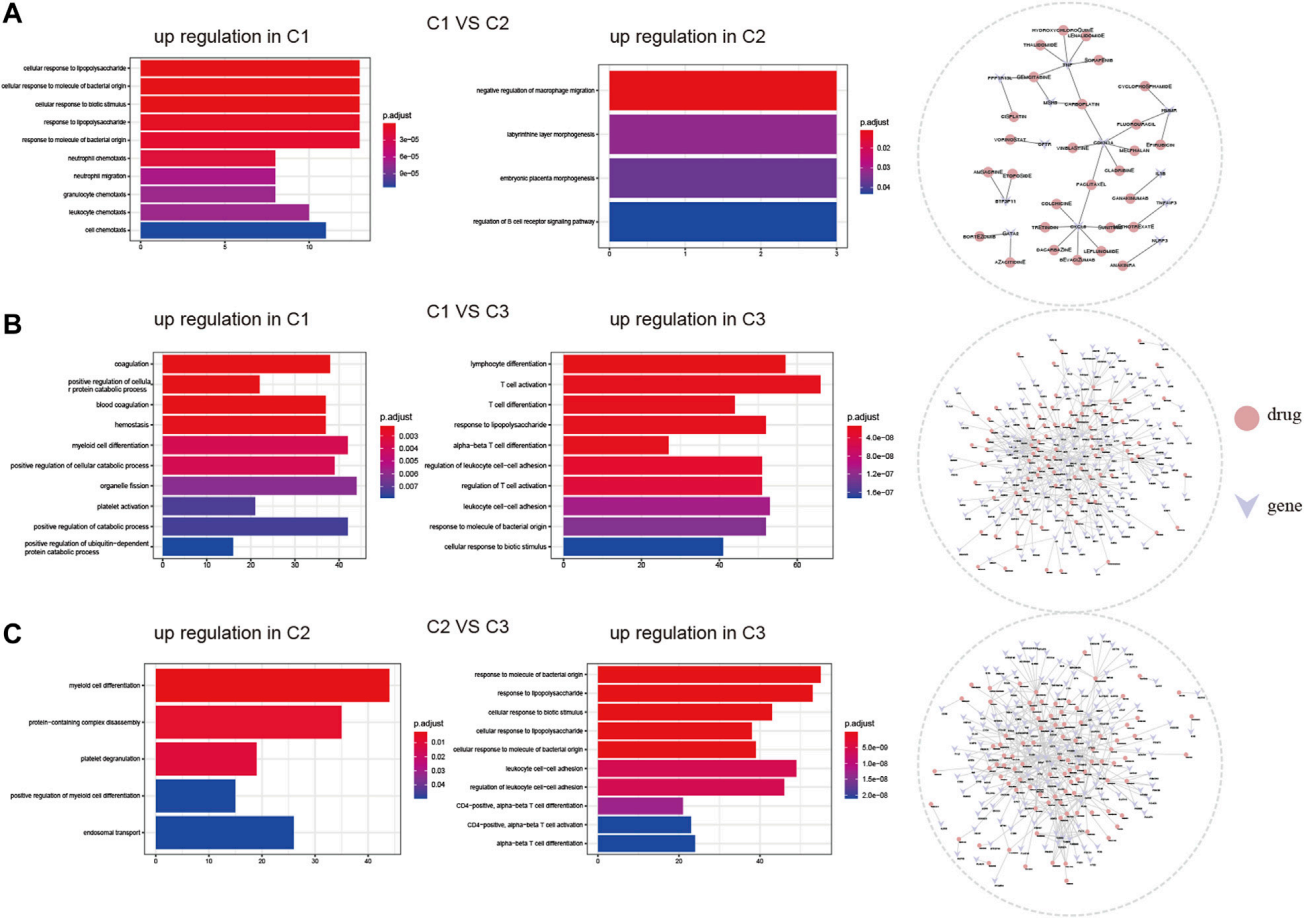
Diversity of underlying biofunctional characteristics among three ischemic stroke subtypes. **(A)**, **(B)**, and **(C)** Upregulated gene enrichment among the three subtypes, pairwise comparisons were made for GO-biological processes, and potential drug targets predicted by DGIdb, respectively.

We investigated the biological responses of the three cell pyroptosis patterns by comparing the DEGs among them and evaluated different subtypes in BP using the clusterProfiler enrichment analysis. The upregulated genes in subtype C1 were more concentrated in neutrophil migration and leukocyte chemotaxis, while those in subtype C2 were more concentrated in the negative regulation of macrophage migration (Figure 6A). The upregulated genes in subtype C1 were more concentrated in coagulation and positive regulation of the cellular protein catabolic process, while those in subtype C3 were more concentrated in T-cell activation and differentiation (Figure 6B). Moreover, the upregulated genes in subtype C2 were more concentrated in myeloid cell differentiation, while those in subtype C3 were more concentrated in response to molecules of bacterial origin and lipopolysaccharide than those of subtype C2 (Figure 6C).

**FIGURE 7:**
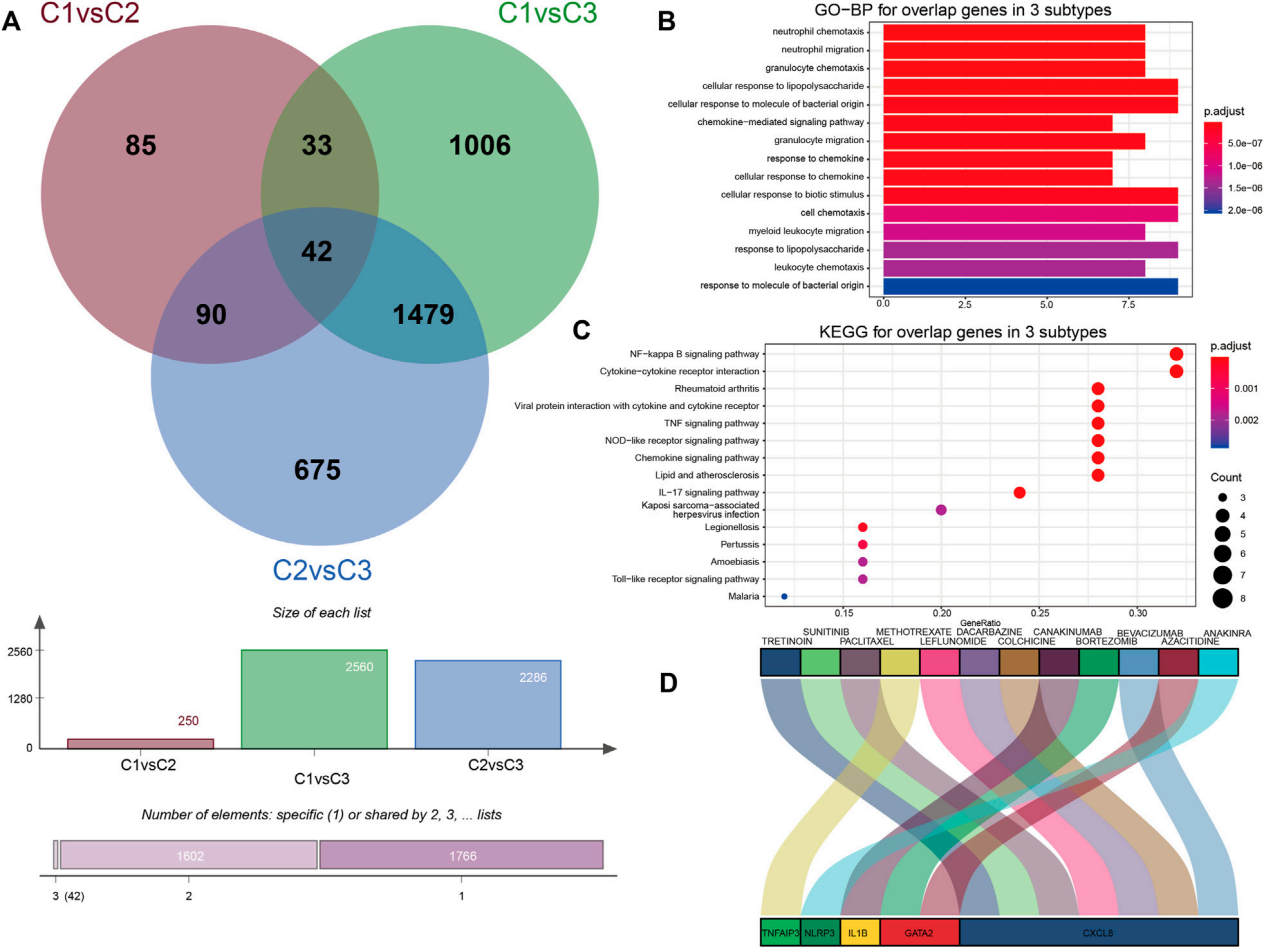
Biological characteristics and potential drugs of three PRG modification patterns. **(A)** Forty-two common genes were identified as genes associated with the pyroptosis phenotype. **(B)** Barplot graph for GO enrichment (the longer bar means the more genes enriched, and the increasing depth of red means the differences were more obvious). **(C)** Bubble graph for KEGG pathways (the bigger bubble means the more genes enriched, and the increasing depth of red means the differences were more obvious; q-value: adjusted *p*-value). **(D)** Sankey diagram showing 42 genes predicted by DGIdb for potential drugs.

The DGIdb provides drug–gene interactions and druggable genome information integrated from over 30 different resources. The drug–gene interaction analysis from DGIdb showed an association of IS-related PRGs and latent target drugs. We identified and overlapped pyroptosis-associated DEGs to obtain genes associated with pyroptosis phenotypes for understanding their molecular mechanisms in pyroptosis regulation further. As shown in Figure 7A, totally 42 common genes were determined to be associated with the pyroptosis pattern phenotype. Then, GO enrichment analysis showed that they primarily took part in leukocyte chemotaxis and reaction to lipopolysaccharides (Figure 7B). KEGG showed that the related pathways involved in these common genes were significantly correlated with the NF-κB (NF-kappa B) signaling pathway and the cytokine–cytokine receptor interaction pathway (Figure 7C). Through the DGIdb, 12 drugs were demonstrated to interact with five genes, which can help develop new treatment methods for IS (Figure 7D). Previous studies showed that IL1B, CXCL8, NLRP3, TNF, and AIP3 participate in pyroptosis and the NF-κB signaling pathway. CXCL8 regulates inflammation through chemotaxis of neutrophils and has a strong pro-angiogenesis effect (He et al., 2018; Lv et al., 2019). Based on the DGIdb results, canakinumab and colchicine were the most noteworthy molecule drugs that were closely associated with IS, and leflunomide and anakinra presented positive anti-inflammatory effects. However, their exact roles in IS remain unclear.

## 4 Discussion

Previous studies have reported that IS involves the activation of immuno-inflammatory responses in systemic inflammation, mainly in chemokine upregulation, peripheral immune cell infiltration, and proinflammatory cytokine release (Tuttolomondo et al., 2009; Masahito and Yenari Midori, 2015; Tomasz, 2015). Pyroptosis, also known as inflammatory necrosis, is an important innate immune response that involves programmed and regulatory cell death. We suspect that pyroptosis might have a paramount effect on shaping IS systemic inflammation because it is indispensable in immune response. At the same time, its specific mechanism and pertinent signaling pathways are unclear (Ye et al., 2020; Liu et al., 2021a). Thus, our study links pyroptosis with systemic inflammation, classifies IS patients into subtypes, and identifies DEGs, signaling pathways, and targeted drugs.

In this study, we investigated the mRNA levels of 33 currently known PRGs in IS and normal samples and found that some of them were differentially expressed. We performed a variety of analyses to elucidate how pyroptosis could influence the immune reactions in IS, enrich infiltrating immunocytes, and activate immune pathways and drew the following conclusions: first, some of the 33 PRGs were found to alter their expressions in IS and healthy samples, including TNF, AIM2, and SCAF11. Many PRGs exhibited expression correlation or protein interaction, which revealed the regulating network of their modification. LASSO and multivariate regression analyses were conducted to construct a classifier based on the three PRGs related to IS and find out which classifier significantly distinguished between healthy and IS samples, suggesting a paramount impact of PRGs on IS development. Second, the correlations between PRGs and immune characteristics in IS were studied, including immune reaction gene sets, HLA gene expression, and infiltrating immunocytes. It was demonstrated that many PRGs were in close association with these immune characteristics, revealing the crucial role of pyroptosis modification in IS systemic inflammation regulation. Post IS, rapid NK cell-mediated exacerbation of brain infarction takes place via the disruption of NK cell tolerance, augmenting local inflammation and neuronal hyperactivity (Gan et al., 2014). NK cells are innate lymphocytes, and their infiltrating abundance is negative with AIM2. Eosinophils significantly predict the severity of acute IS (Wang et al., 2017), and infiltrating eosinophil abundance is positively associated with IL1B. In addition, infiltrating mast cell abundance is positively associated with TNF that mediates blood–brain barrier disruption in IS (Mattila Olli et al., 2011). We found that AIM2 was negatively correlated with cytokine receptors and SCAF11 was negatively correlated with cytokine activity. NLRP3 and TNF were positively correlated with antimicrobials and chemokines, respectively. Cytokines and chemokines are important components of innate immunity and have a paramount impact on IS (Sen et al., 2007; Carlo Domenico. et al., 2020).

During the unsupervised clustering of IS samples based on gene expression related to pyroptosis, we found three subtypes with distinctive PRGs and unique immune characteristics. CD4^+^ T lymphocytes are crucial mediators of IS tissue damage and can inhibit B-cell infiltration into the brain (Weitbrecht et al., 2021). The results revealed that CD4^+^ T-cell activation was the highest in subtype C3 but the lowest in subtype C2. The consensus clustering analysis of the three subtypes showed significant differences between gender and alcohol consumption in clinical characteristics. Next, the biological responses and related signaling pathways of the three cell pyroptosis modes were studied. The results of the GO analysis showed that the 42 common genes associated with the cell pyroptosis phenotype primarily took part in the chemotaxis of leukocytes and reaction to lipopolysaccharides, indicating that pyroptosis induced extensive inflammatory responses. The NF-κB signaling pathway as a positive transcriptional regulator of GSDMD was identified via KEGG analysis (Pickering Robert and Bryant Clare, 2020). GSDMD is the executive protein and characteristic biomarker of pyroptosis (Liu et al., 2017).

Subsequently, the obtained IS genes were combined with existing drugs through drug–gene interactions for analysis and exploring latent immunotherapeutic targets for IS. Based on the DGIdb online tool searching for targeted drugs, we predicted 12 small molecules as possible drugs for IS treatment, which potentially target IL1B, CXCL8, NLRP3, TNF, AIP3, and GATA2 genes. The last two promising drugs contained canakinumab and colchicine and have been tested via clinical trials. Canakinumab is regarded as a latent drug for IS because of its inhibition of IL-1β. Recent studies confirmed that canakinumab specifically reduces IL-1β-mediated inflammatory lesions in cerebrovascular diseases (Luca. et al., 2018; Fatemeh et al., 2021; Sjöström Elisabet et al., 2021). Similar to IL-1β monoclonal antibodies, canakinumab has been approved for several auto-inflammatory disorders, including classic SJIA (systemic juvenile idiopathic arthritis), gout, and macrophage activation syndrome. Colchicine was regarded as a latent drug for IS because it inhibited CXCL8. Colchicine has an anti-inflammatory function and can relieve ongoing tissue damage caused by neutrophils, NK cell migration and activation, and inflammatory cytokine release (He et al., 2018; Ami, 2021). These findings provide a theoretical basis for the development of latent therapeutic drugs.

This research was the first to systematically analyze the relationship between PRG regulation and systemic inflammation in IS. In addition, three distinct pyroptosis expression patterns that differed from other classification standards in IS were identified. These results could greatly guide immunotherapy development with respect to pyroptosis in IS and provide researchers with a direction to implement these studies. However, there are some limitations to this research design. First, our research primarily focuses on bioinformatics analysis based on numerous previous pyroptosis research studies; therefore, *in vitro* and *in vivo* experiments are still required to verify these results. Second, the analysis of immune cells mainly used the bioinformatics analysis method to evaluate immunocyte quantity, but the most reliable counting method is single-cell sequencing. The results of single-cell sequencing can possibly explain the specific changes in the IS systemic inflammation. We will address this concern in further studies. Moreover, in this study, some of the mRNAs identified may be false-positives owing to the small number of samples from IS patients. In further studies, we will use a greater number of samples to confirm their *in vivo* roles in IS.

In conclusion, the research demonstrates the latent regulation mechanisms of PRG modification in IS systemic inflammation. The diversity of PRG modification patterns has a crucial effect on the complexity and heterogeneity of IS systemic inflammation. In our study, the integrated analysis of PRG modification patterns is conducive to investigating the immune-regulated network mechanism and exploring more effective immune-related therapies in IS.

## Data Availability

The datasets presented in this study can be found in online repositories. The names of the repository/repositories and accession number(s) can be found in the article/Supplementary Material.

## Abbreviations

IS, ischemic stroke; PRGs, pyroptosis-related genes; DEGs, differentially expressed genes; GSDMD, gasdermin-D; GSDME, gasdermin-E; NT, N-terminal; CT, C-terminal; IL-18, interleukin-18; IL-1β, interleukin-1β; NK cell, nature killer cell; NF-κB, NF-kappa B; GEO, Gene Expression Omnibus; PPI, protein–protein interaction; STRING, Search Tool for the Retrieval of Interacting Genes; PCC, Pearson correlation coefficient; LASSO, least absolute shrinkage and selection operator; ROC, receiver operating characteristic; ssGSEA, single-sample gene set enrichment analysis; HLAs, human leukocyte antigen genes; PCA, principal component analysis; GO-BP, Gene Ontology-biological process; KEGG, Kyoto Encylopedia of Gene and Genomes; DGIdb, Drug–Gene Interaction Database; SJIA, systemic juvenile idiopathic arthritis.
