# Supplementary figures and images for: Identification of pyroptosis-related immune signature and drugs for ischemic stroke
Source: Front Genet. 2022 Sep 27;13:909482. doi: 10.3389/fgene.2022.909482 (PMC9552296; doi:10.3389/fgene.2022.909482)

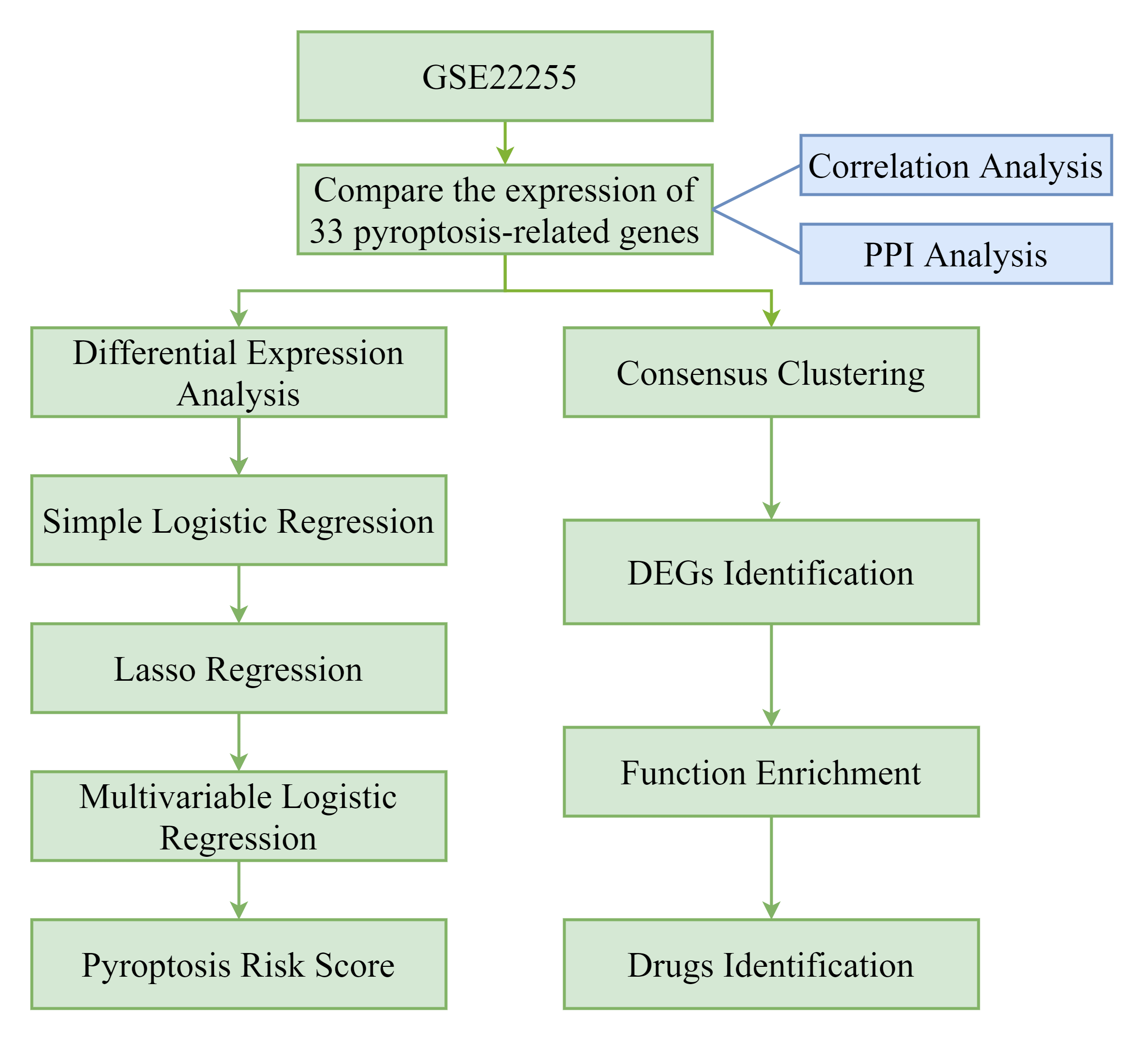

Supplement: Supplementary file 2 [file DataSheet2.zip › supplementary figures/Supplementary Figure S1.TIF]

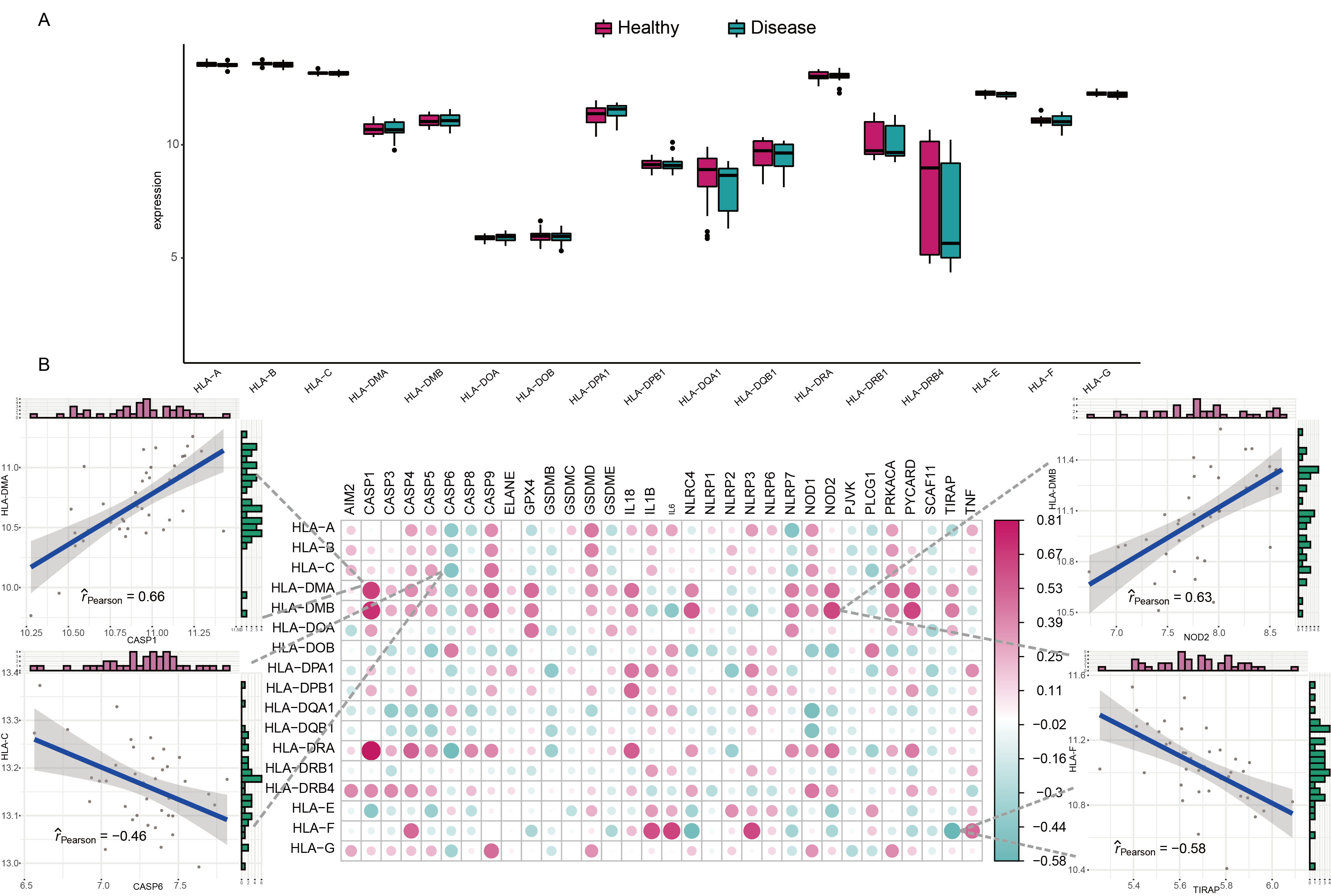

Supplement: Supplementary file 2 [file DataSheet2.zip › supplementary figures/Supplementary Figure S2.TIF]

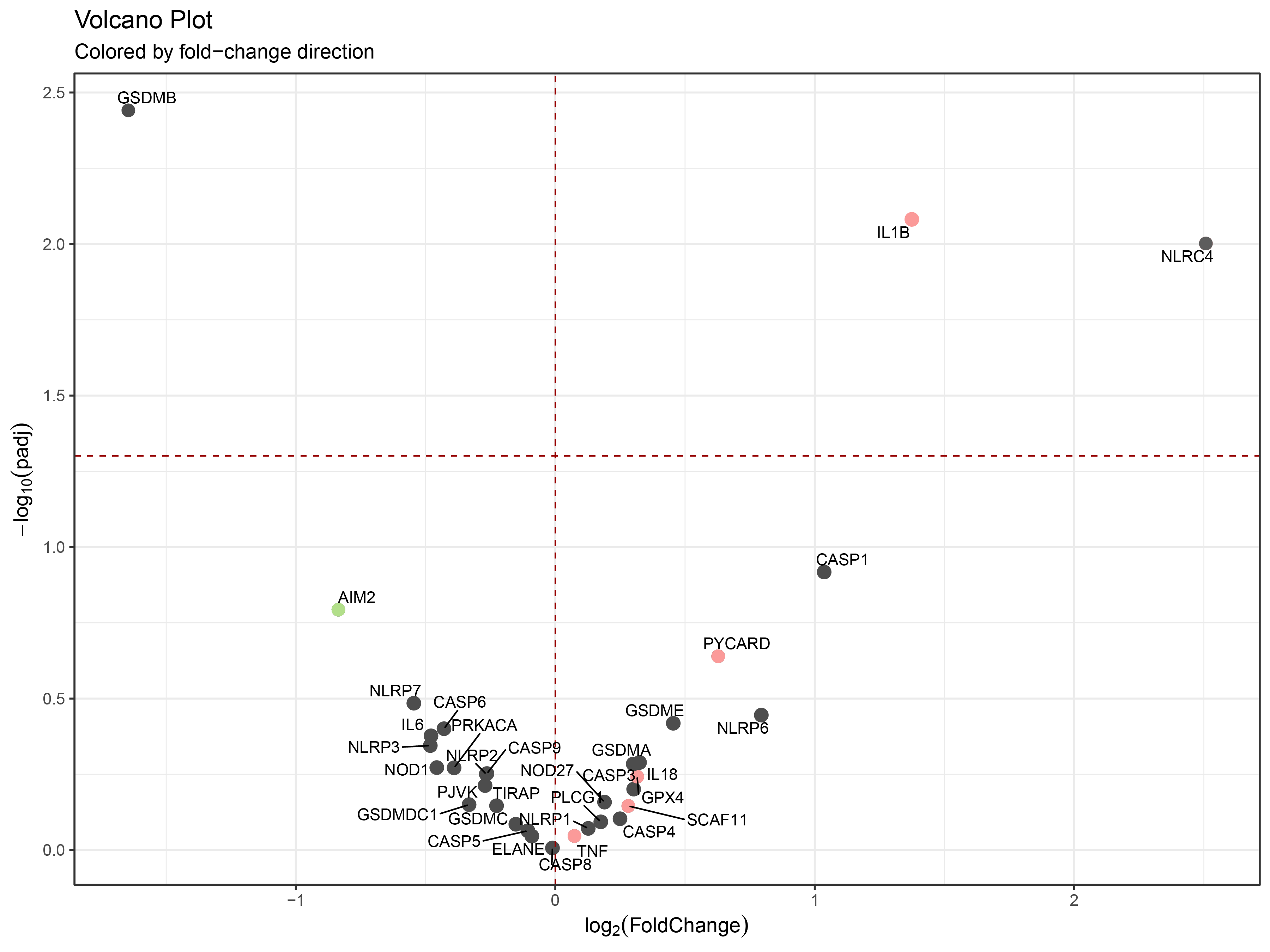

Supplement: Supplementary file 2 [file DataSheet2.zip › supplementary figures/Supplementary Figure S3.TIF]

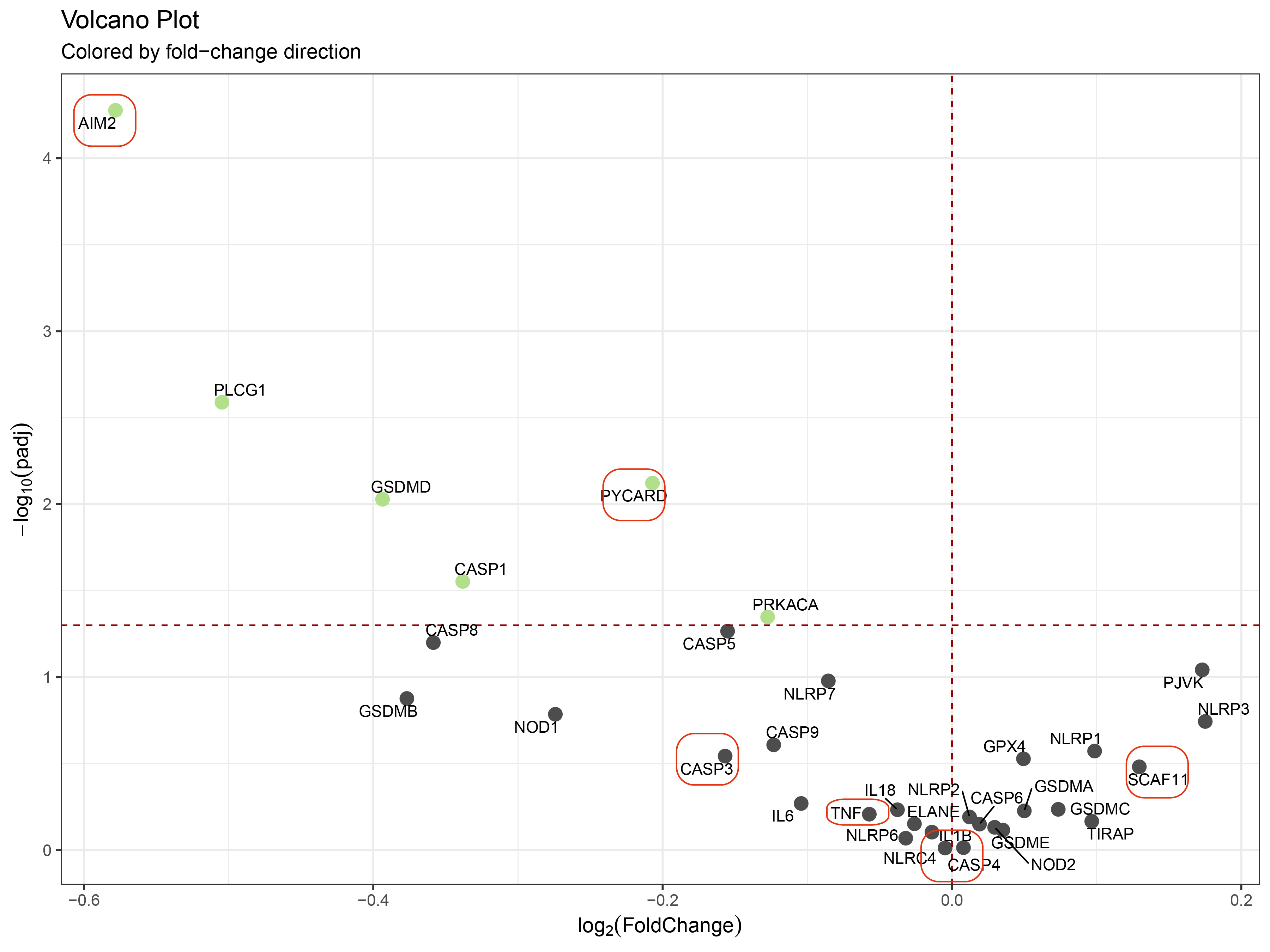

Supplement: Supplementary file 2 [file DataSheet2.zip › supplementary figures/Supplementary Figure S4.TIF]
