# Supplementary material for: Identification of pyroptosis-related immune signature and drugs for ischemic stroke
Source: Front Genet. 2022 Sep 27;13:909482. doi: 10.3389/fgene.2022.909482 (PMC9552296; doi:10.3389/fgene.2022.909482)
Supplement: Supplementary file 1 [file DataSheet1.ZIP › table/Original data Description.doc]

GSE22255_geneexpr.txt：GSE22255 standardized expression profile

GSE22255-clinical.txt：GSE22255 clinical information

difall_GSE22255.csv：Differential expression results

logistic.txt：Univariate Logistic results

ssGSEA_output_group.txt ：Infiltration of each sample in SSGSEA

Immport_GeneList.txt HLA ：Immune response gene set response score:

HLA_expr.txt；Gene set-sample expression profile

GSE22255-cluster3.txt：ConsensuClusterPlus typing results

different expressed gene in subtype.xlsx：Differentially expressed genes between subtypes

DGIdb.xlsx：Differentially expressed gene-drug targeting relationships among subtypes

venn3type.txt：Three differentially expressed genes of Overlop

dgidb_C1vsC2vsC3.tsv：42 core gene-drug targeting relationships
